# Bacteria

**Published:** 1883-01

**Authors:** J. M. Adams


### BACTERIA.

Bacteria, whether significant of disease or decline of health, are found more or less numerous in everything we eat and drink. The germs or spores of many kinds, known as *termo*, *lineola*, tenue, spirillum, vibriones, etc., exist in almost infinite numbers; some of the smallest are too small to be seen by the highest powers, which, being lodged in all vegetable and animal substances, spring into life and develop very rapidly under favorable circumstances. They develop most rapidly when decomposition commences, and seem to indicate the degree or activity of that decomposition, also hastening the same. They are found most numerous in the fæces, and usually fully developed in the fresh evacuations of persons of all ages. They may be seen plainly under a thin glass with high powers, with strong or clear light, when the material is much diluted with water.

These bacteria appear almost as numerously, yet more slowly, in urine, either upon exposure to air or when freshly evacuated, when the general health of the individual is declining, or any tendency to decomposition. A diagnosis can be aided very greatly by a study of these bacteria, as they indicate or determine the vitality, vigor and purity of the system, whether more or less subject to disease, even before any signs of disease appear. They seem to preindicate the hold of the life force on the material, and always appear when that force is broken. Their relative quantity found in fæces is as a barometric indication of the general health or some particular disturbance, and it is surprising how very fast they multiply while simply passing the intestines under circumstances favorable for their growth. These forms, so small, are important, because so very numerous, and their study has been, perhaps, avoided by many; yet they certainly mean something and effect something, even the non-malignant varieties as mentioned above, and it is certainly worth while to continue to study their meaning, even beyond what has already been written by others on the subject.—*J. M. Adams in the Microscope.*

